# Integrated Transcriptomic Analysis of the miRNA–mRNA Interaction Network in Thin Endometrium

**DOI:** 10.3389/fgene.2021.589408

**Published:** 2021-03-16

**Authors:** Lu Zong, Shengxia Zheng, Ye Meng, Wenjuan Tang, Daojing Li, Zhenyun Wang, Xianhong Tong, Bo Xu

**Affiliations:** Reproductive and Genetic Hospital, The First Affiliated Hospital of USTC, Division of Life Sciences and Medicine, University of Science and Technology of China, Hefei, China

**Keywords:** thin endometrium, transcriptome analysis, miRNA, mRNA, regulatory

## Abstract

Although the thin endometrium (TE) has been widely recognized as a critical factor in implantation failure, the contribution of miRNA–mRNA regulatory network to the development of disease etiology remains to be further elucidated. This study performed an integrative analysis of the miRNA–mRNA expression profiles in the thin and adjacent normal endometrium of eight patients with intrauterine adhesion to construct the transcriptomic regulatory networks. A total of 1,093 differentially expressed genes (DEGs) and 72 differentially expressed miRNAs (DEMs) were identified in the thin adhesive endometrium of the TE group compared with the control adjacent normal endometrial cells. Gene Ontology (GO) and Kyoto Encyclopedia of Genes and Genomes (KEGG) pathway analyses showed that the DEGs and the target genes of DEM were significantly enriched in angiogenesis, cell growth regulation, and Wnt signaling pathway. Multiple hub genes (CAV1, MET, MAL2, has-mir-138, ARHGAP6, CLIC4, RRAS, AGFG1, has-mir-200, and has-mir-429) were identified by constructing the miRNA–mRNA regulatory networks. Furthermore, a miRNA–mRNA pathway function analysis was conducted, and the hub genes were enriched in the FoxO signaling pathway, cell growth regulation, inflammatory response regulation, and regulation of autophagy pathways. Our study is the first to perform integrated mRNA-seq and miRNA-seq analyses in the thin adhesive endometrium and the control adjacent normal endometrial cells. This study provides new insights into the molecular mechanisms underlying the formation of thin endometrium.

## Introduction

The endometrium is an indispensable factor for implantation and pregnancy, and an increase in endometrial thickness promotes an increased pregnancy rate. An endometrial thickness of <7 mm is usually regarded as sub-optimal for embryo transfer and results in a decreased probability of pregnancy (Shufaro et al., 2008). For patients with Asherman's syndrome (AS), repetitive curettage or invasive endometritis disrupts endometrial regeneration, thus resulting in a fibrotic and thin endometrium (TE) (Azizi et al., 2018). Patients suffering from thin or fibrotic endometrium are more susceptible to abnormal menstruation and, particularly, fertility impairments, such as a decreased pregnancy rate, unfavorable pregnancy outcomes, or recurrent pregnancy loss (Du et al., 2020). The current AS treatments aim to increase endometrial regeneration with low-dose aspirin, exogenous estrogen, vitamin E, vaginal sildenafil citrate, cytokines, and colony-stimulating factors (CSFs). Nonetheless, these treatments are unable to attain a satisfactory clinical response in many patients with TE (Azizi et al., 2018). The definite etiology and physiopathology of thin endometrium remain largely unclear at present. Therefore, studies aiming to explore the related molecular mechanism of TE are urgently needed to guide disease therapy in the future.

A transcriptomic analysis is essential for understanding the occurrence and pathogenic mechanism of thin endometrium. Only one existing study has reported the global transcriptomic abnormalities in thin endometrium at the mid-luteal phase (Maekawa et al., 2017). The study compared the transcriptomic profiles between three patients and three normal subjects using the Gene Chip Human Genome U133 Plus 2.0 Array platform. Finally, 318 genes were upregulated in the thin endometrium, while 322 genes were downregulated. According to that study, implantation failure induced by thin endometrium might be related to the abnormal activation of the inflammatory environment, together with an abnormally reduced oxidative stress (OS) response. Nonetheless, researchers have not clearly elucidated the underlying mechanism of endometrial regeneration dysfunction in patients with thin endometrium. Additionally, more studies are needed to comprehensively characterize how thin endometrium affects the transcriptomic profiles.

MicroRNAs (miRNAs) are non-protein-coding RNA molecules with short (20–25) nucleotides. miRNAs bind to target mRNAs for transcription and translation regulation, including mRNA degradation, cleavage, or translational repression (Shukla et al., 2011; Li et al., 2019). miRNAs have been deemed to participate in the regulation of various cellular processes, including cellular proliferation, differentiation, apoptosis, and angiogenesis (Laurent, 2008; Nicoli et al., 2010; Hong et al., 2019). Recently, more and more miRNAs were found to be associated with endometrial receptivity (Altmae et al., 2013), endometrial stromal cell differentiation (Qian et al., 2009), embryo development (Laurent, 2008), and implantation (Paul et al., 2019). The expression of miR-27a-3p and miR-124-3p was downregulated in the endometrium of chronic endometritis (Di Pietro et al., 2018). The expression of hsa-miR-449a, hsa-miR-3135b, and hsa-miR-345-3p could promote endometrium receptivity in preparation for *in vitro* fertilization and embryo transplantation (Mu et al., 2020). miR-30 and miR-200 family members have been repeatedly recognized as important miRNAs in the regulation of endometrial receptivity (Rekker et al., 2018). Aberrant miR-200 expression may negatively regulate endometrial development and decidualization (Jimenez et al., 2016) and plays an important role in regulating normal endometrial development and disorders such as endometriosis and endometrial cancer (Panda et al., 2012). However, few studies have investigated the effect of miRNA on thin endometrium. The dysfunction of endometrium cells in TE and how miRNAs regulate the pathogenesis of TE remain to be elucidated.

Our article aimed to identify the miRNA–mRNA networks and molecular pathways in women experiencing intrauterine adhesion (IUA) and to provide additional insights into the underlying transcriptomic mechanisms by performing RNA-Seq. The differentially expressed miRNA–mRNA regulatory axis along with the gene pathway–function network interactions in thin endometrium was constructed. Our findings supply a basis to better investigate the biological mechanisms of thin endometrium and facilitate the formulation of molecular targeted treatments for thin endometrium.

## Materials and Methods

### Tissue Sample Collection

Eight females aged 20–40 years old, with a history of severe IUAs (Grade III–V) as diagnosed by hysteroscopy at the Reproductive Medicine Center of The First Affiliated Hospital of the University of Science and Technology of China, were enrolled in the study. The severity of IUAs was determined according to the American Fertility Society classification system (1988 version) (1988). Scores of 9–12 represented severe adhesions. The thickness of the endometrium was determined through vaginal ultrasound (at mid-luteal phase) as the maximum distance between endometrial interfaces, and the endometrial thickness in all patients was <7 mm. The sample information is described in Table 1. The endometrial tissue from the IUA (TE group) and adjacent normal endometrium tissues (AJ-CN group) from eight patients with severe IUAs (Grade III–V) were analyzed in the present study. This study was approved and monitored by the Human Research Ethics Committee of the First Affiliated Hospital of the University of Science and Technology of China. Each patient was required to provide a written informed consent prior to participation in this study. Endometrial tissues were sampled at mid-luteal phase during the menstrual cycle. Afterwards, the collected endometrial tissue samples were rinsed with saline to remove blood and then stored in liquid nitrogen at −80°C until subsequent RNA isolation.

**Table 1:** Information about the sample of patients with thin endometrium analyzed in our study.

**Group**	**Age (years)**	**Endometrial thickness (mm)**	**Sample date (from LMP)**	**History of gestation**	**Score of IUA**
TE 1	31	6.5	20	G3P1	9
TE 2	28	5.6	21	G3P1	10
TE 3	35	4.8	22	G2P1	10
TE 4	32	5.2	21	G2P1	11
TE 5	27	6.4	21	G2P1	10
TE 6	32	5.8	20	G4P1	12
TE 7	37	6.5	22	G3P1	10
TE 8	30	5	21	G2P1	10

*LMP, last menstrual period; TE, thin endometrium; history of gestation, G for gestation, P for parturition*.

### RNA Isolation and Library Construction

Total tissue RNA was extracted using TRIzol reagent (Invitrogen, Carlsbad, CA, USA), pooled equally, and reverse-transcribed into cDNAs using the QuantiTect Reverse Transcription Kit (Qiagen, Valencia, CA, USA) according to the manufacturer's specific instructions. The quantity and quality of the extracted RNA were measured using Nanodrop (Thermo Scientific). The cDNA library was constructed using KAPA Stranded RNA-Seq Library Preparation Kit (Illumina) following the manufacturer's protocol. The synthetic cDNAs were end-repaired by polymerase and ligated with “A-tailing” base adaptors. Suitable fragments were selected for polymerase chain reaction (PCR) amplification to construct the final cDNA library. The final double-stranded cDNA samples were verified with Agilent 2100 Bioanalyzer (Agilent Technologies). Sequencing was performed on an Illumina HiSeq 4000 sequencing platform with 150-bp paired-end sequencing.

Then, the combined RNA samples were separated using 15% (w/v) denaturing polyacrylamide gel electrophoresis. Subsequently, miRNA fragments with a size of ~18–28 nt were separated by gel extraction, followed by RNA purification. The total RNA of each sample was used to prepare the miRNA sequencing library, which included the following steps: (1) 3′-adaptor ligation, (2) 5′-adaptor ligation, (3) cDNA synthesis, (4) PCR amplification, and (5) size selection of ~135–155-bp PCR-amplified fragments (corresponding to ~15–35 nt small RNAs). Libraries were quantified and validated with Agilent 2100 Bioanalyzer (Agilent Technologies). Thereafter, the small RNA library was sequenced using Illumina Hiseq 4000 (Illumina, San Diego, CA, USA), with a configuration of 50 cycles single reads according to the manufacturer's recommendations. All sequencing procedures were performed by Kang Chen Bio-tech (Shanghai, China).

### mRNA Sequencing and Data Analysis

Raw data were pre-processed using Solexa CHASTITY and Cutadapt to remove adaptor sequences, ribosomal RNA, low-quality reads, and other contaminants that may interfere with assembly. The criteria for this filtering procedure were set as follows: (1) RNA 5′ and 3′ adapters were removed, respectively, (2) bases with a phred quality score below 20 were clipped from both ends of reads, (3) after low-quality bases were trimmed, reads containing over two “N” were discarded, (4) reads with a length shorter than 75 nt were discarded; and (5) the parameters for BWA v0.5.724 were set as recommended according to Fastq_clean instructions. Then, the sequence quality was examined using FastQC v0.11.7. Afterwards, Hisat2 was utilized to align those trimmed reads to the reference genome. StringTie (version 1.2.3) was used to reconstruct the transcriptome. Fragments per kilobase per million (FPKM) values of genes were normalized with Ballgown using the default parameters. FPKM ≥0.5 (Cuffquant) was considered as statistically significant for the next DEG analysis. RNA sequencing data were deposited into the Gene Expression Omnibus (GEO accession number GSE160635).

### miRNA Sequencing and Data Analysis

The miRNA sequencing data from TE group and AJ-CN group endometrium cells were analyzed by our previously published tool, DeAnnIso (Zhang et al., 2016). Briefly, after sequencing, Bowtie was used for mapping reads into the reference genome. The aligned reads had no more than “N” mismatches (0–3, default is 2) in the first “L” bases (≥5, default is 10) of the left end. Thereafter, those precursor sequence-matched reads were aligned to the pooled pre-miRNA databases (known pre-miRNAs in miRBase v21) using the BLAST. The default E-value was set to 0.01 for BLAST. All the detected isomiRs were aligned with their canonical miRNAs, the numbers of mapped reads that were defined as the raw expression levels of that miRNA. To correct for the difference in read counts between samples, the read counts were scaled to reads per million (RPM). Small RNA sequencing data were deposited into the Gene Expression Omnibus (GEO accession number GSE108966).

### Differential Expression Analysis

After excluding the transcripts with a low count, genes with an FPKM or RPM ≥5 in at least one sample were used for the analysis. Fold change (FC) and *P*-value for Fisher's exact test was calculated and used when comparing the differentially expressed mRNAs (DEGs) and miRNAs (DEMs) between the two groups. The log_2_FC derived from the comparisons of the FPKM or RPM values of the TE group with the AJ-CN group is depicted (|Log_2_FC| ≥ 2) and *P* < 0.05 were selected as the cutoff criteria to identify significant DEMs and DEGs. Additionally, TargetScan (Garcia et al., 2011) and miRDB (Wang and El Naqa, 2008) were used to predict mRNAs targeted by DEMs.

### Functional Annotations

Gene Ontology (GO) and Kyoto Encyclopedia of Genes and Genomes (KEGG) pathway enrichment analyses were performed using the online analysis tool of Annotation Visualization and Integrated Discovery (https://david.ncifcrf.gov/). The *P*-value for Fisher's exact test was calculated as a result of enrichment degree. GO term enrichment of biological processes or KEGG pathway annotations with a *P*-value cutoff of 0.05 were identified as an important term in this study.

### Construction of the Protein–Protein Interaction Network

The Search Tool for the Retrieval of Interacting Genes (STRING) database (http://www.string-db.org/) was used to construct the PPI network. The obtained interactions included both the known and the estimated interactions. A requisite confidence value (pooled score >0.4) was used as the threshold. In addition, Cytoscape v3.7.1 was utilized to visualize the PPI network, and CytoHubba functions were employed to identify the hub genes. Genes with Gene significance >0.2, module membership >0.8, and *P* ≤ 0.05 were defined as hub genes.

### Construction of the DEM–DEG Regulatory Network

TargetScan (http://www.targetscan.org/) and miRDB (http://www.mirdb.org/) were utilized to preliminarily predict DEM target genes. The co-predicted targets were used for further GO and KEGG pathway enrichment analyses. The genes shared between DEM targets and DEGs were used to analyze the miRNA–mRNA pairs, which were maintained to construct the DEM–DEG regulatory network with Cytoscape. Differentially expressed target genes were chosen for GO and KEGG pathway analyses to investigate the miRNA–mRNA regulatory networks in TE.

## Results

### Genome-Wide Patterns of the mRNA Transcriptomic Landscape

Using Illumina Hiseq 4000, 18,354,811 original RNA reads were obtained from the thin endometrial cells of patients with IUA, and 21,755,164 reads were obtained from adjacent normal endometrial cells. After removing adaptor sequences and low-quality reads, 18,288,140 (thin endometrial cells from patients with IUA) and 21,745,564 (adjacent normal endometrial cells) clean reads remained. Then, the genes were normalized to FPKM, and 15,561 genes were expressed in endometrial tissues from those eight women.

**Figure 1:**
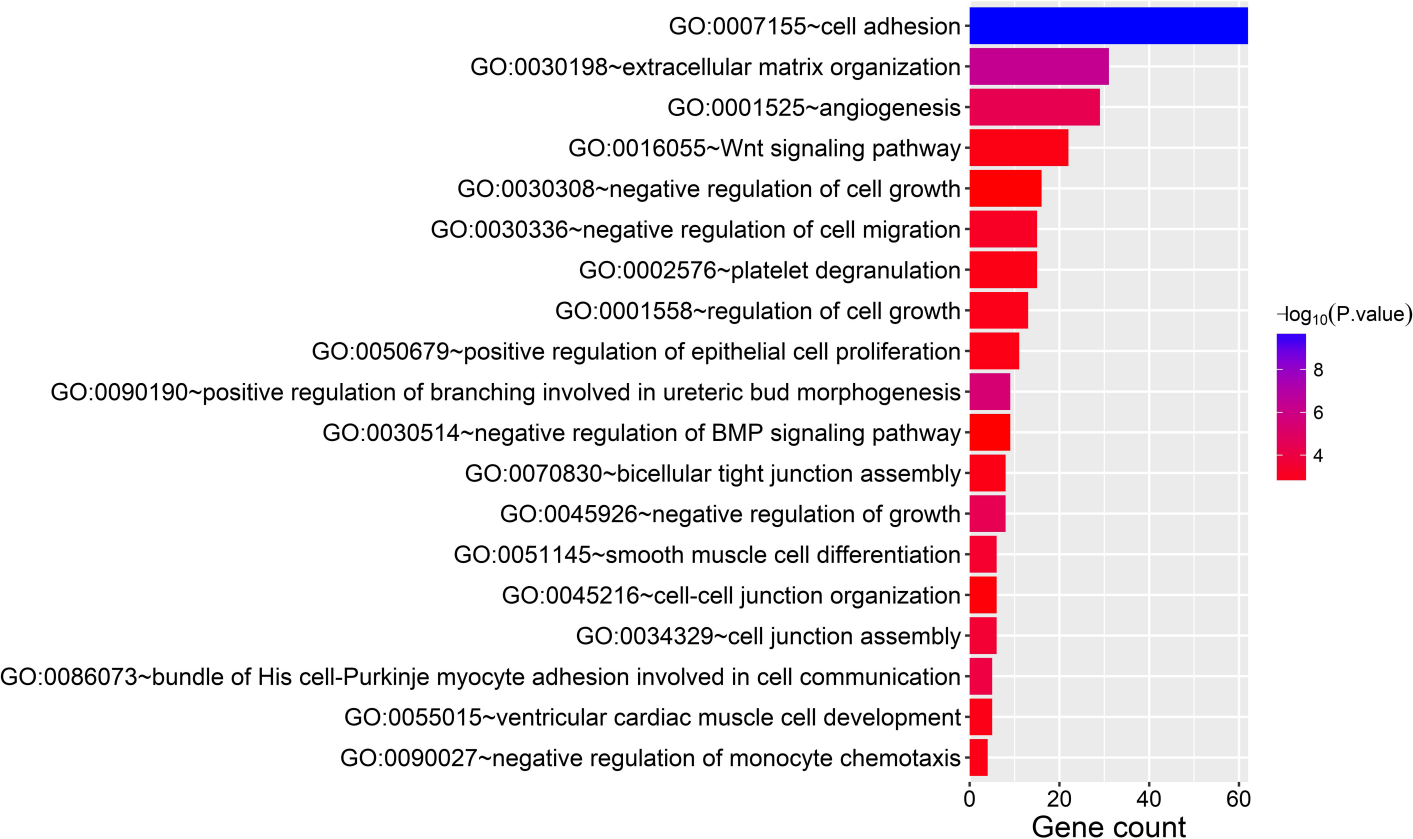
Gene Ontology analysis of 1,093 differentially expressed genes between thin endometrium and adjacent normal endometrium.

**Figure 2:**
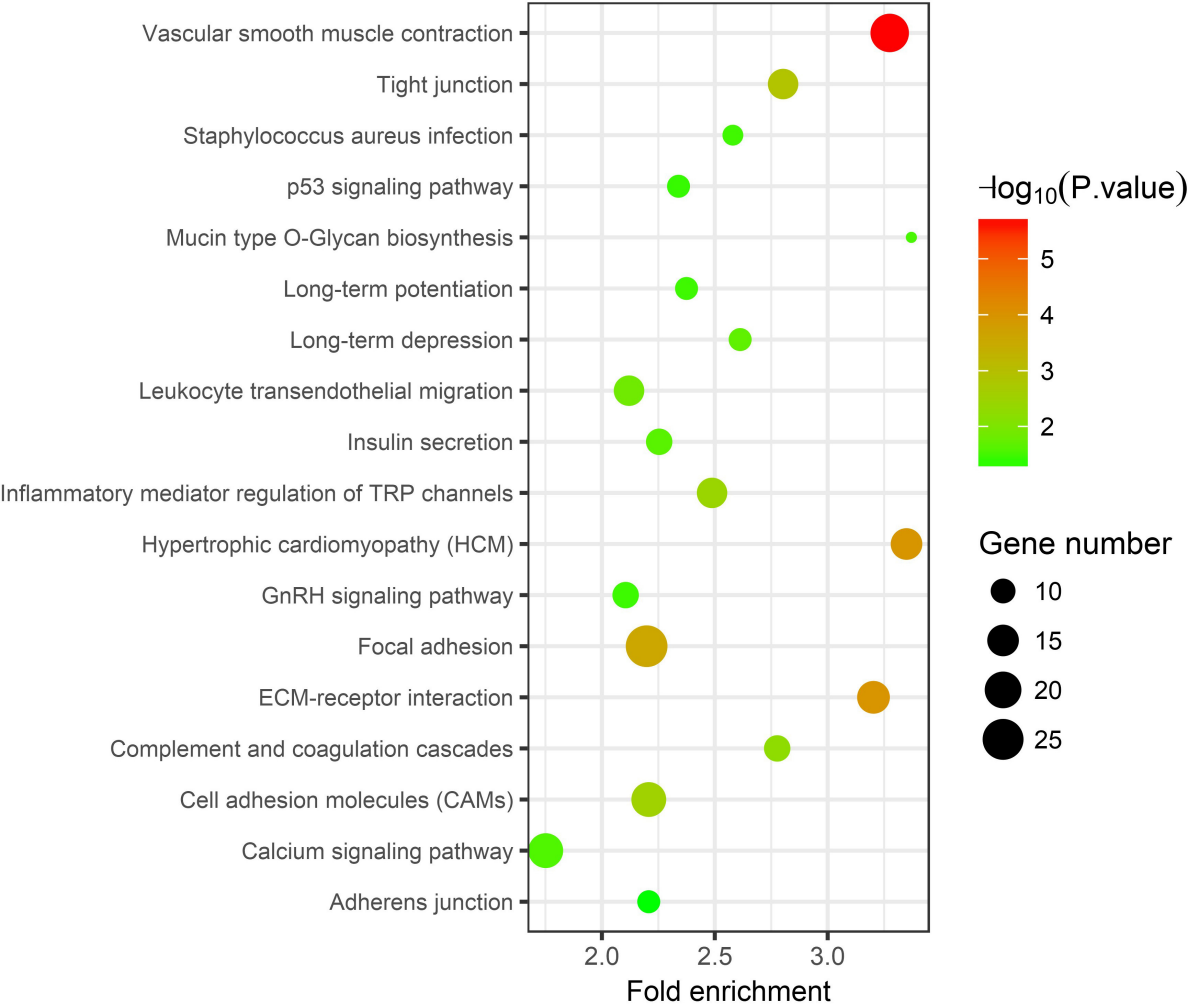
Kyoto Encyclopedia of Genes and Genomes pathway analysis of 1,093 differentially expressed genes between thin endometrium and adjacent normal endometrium.

In the thin adhesive endometrial tissue of the TE group, 374 genes were upregulated, while 719 genes were downregulated compared to the control adjacent normal endometrial cells (Supplementary Tables 1, 2). The GO analysis of 1,093 DEGs identified many genes that were significantly enriched in the cell adhesion process (GO: 0007155, *P* = 1.92E-10), negative regulation of growth (GO: 0045926, *P* = 4.46E-05), angiogenesis (GO: 0001525, *P* = 4.63E-05), cell junction assembly (GO: 0034329, *P* = 2.99E-04), negative regulation of cell migration (GO: 0030336, *P* = 7.39E-04), the Wnt signaling pathway (GO: 0016055, *P* = 0.0017), and negative regulation of the BMP signaling pathway (GO: 0030514, *P* = 0.003) (Table 2 and Figure 1). A blockade angiogenesis was considered as the main pathological change in the scarred thin endometrium (Jiang et al., 2019). Moreover, this study identified several DEGs-related signaling pathways by performing KEGG pathway enrichment analysis, including the vascular smooth muscle contraction pathway, extracellular matrix–receptor interaction, focal adhesion, tight junction, cell adhesion molecules, calcium signal transduction pathway, p53 signal transduction pathway, and adherens junction pathway (Table 3 and Figure 2). The 1,093 DEGs were also compared with the primary associated changes identified in the transcriptome of the thin endometrium (Maekawa et al., 2017), and nine commonly upregulated genes (PDLIM3, FABP3, HIF3A, FILIP1, DPP6, MYOCD, PRKCB, ALDH1B1, and TRNP1) and 65 commonly downregulated genes were identified (Supplementary Table 3). The expression of MYOCD (myocardin), a cardiac-specific co-activator of serum response factor, was upregulated in thin endometrium, while ADAM12 (a disintegrin and metalloproteinase 12) expression was decreased in thin endometrium, and these genes are associated with the fibrosis process (Li et al., 2018; Mittal et al., 2019; Nakamura et al., 2019).

**Table 2:** Gene Ontology analysis of the 1,093 differentially expressed genes between thin endometrium and adjacent normal endometrium.

**Term**		**Count**	***P*-value**	**Genes**
GO:0007155	Cell adhesion	62	1.92E-10	NRP2, MPZL3, CXCL12, PRKX, HMCN2, AZGP1, WISP2, WISP1, CTGF, COL12A1, AFDN, CEACAM1, EGFL6, ADGRE5, NECTIN4, GRHL2, CTNNA2, JUP, NCAM1, PGM5, CD36, LAMC3, VCAN, LAMC2, TGFB1I1, MFAP4, ADAM12, AOC3, OLFM4, ITGA11, PCDHGC3, ALCAM, LAMB3, SORBS1, ITGB8, COMP, MSLN, THBS1, ENTPD1, DPT, SPP1, HAPLN2, SELP, LPP, MCAM, EMILIN2, TINAGL1, COL4A6, LAMA2, ITGA9, CDH13, NME1-NME2, CDH16, DSG2, FREM2, CDON, ITGA7, NLGN4X, DSC2, PDZD2, OMG, MUC16
GO:0030198	Extracellular matrix organization	31	4.19E-07	MPZL3, ELF3, PDGFA, NPNT, ITGA11, CDH1, SOX9, SMOC2, LAMB3, HPSE, ITGB8, COMP, THBS1, SPP1, RXFP1, EGFL6, CCDC80, SPINT1, OLFML2A, COL4A6, COL4A5, LAMA2, ITGA9, BGN, LAMC3, KAZALD1, FBLN5, ITGA7, LAMC2, VCAN, MFAP5
GO:0090190	Positive regulation of branching involved in ureteric bud morphogenesis	9	3.85E-06	NOG, AGTR2, HOXB7, PAX8, SIX4, PAX2, GREM1, SOX9, WNT2B
GO:0045926	Negative regulation of growth	8	4.46E-05	HIF1A, MT1M, MT2A, MT1H, MT1X, MT1G, MT1F, IGFBP5
GO:0001525	Angiogenesis	29	4.63E-05	NRP2, SAT1, CAV1, HTATIP2, PDGFA, CSPG4, FGF10, PRKX, NOV, TYMP, OVOL2, UNC5B, CTGF, XBP1, HS6ST1, SOX17, RAMP1, CEACAM1, SCG2, KLF5, MCAM, ECM1, HOXB3, HIF1A, CLIC4, ID1, PROK1, HIF3A, RBPJ
GO:0086073	Bundle of His cell-Purkinje myocyte adhesion involved in cell communication	5	1.30E-04	JUP, DSG2, PKP2, DSC2, DSP
GO:0034329	Cell junction assembly	6	2.99E-04	LIMS2, FERMT2, ILK, FLNC, GRHL2, FLNA
GO:0051145	Smooth muscle cell differentiation	6	4.63E-04	MEF2C, WNT4, MYOCD, GATA6, HEY2, FGF10
GO:0030336	Negative regulation of cell migration	15	7.39E-04	PTPRJ, NOG, EPPK1, PLXNB3, DPYSL3, SLC9A3R1, TPM1, SLIT2, WNT4, PKP2, CLIC4, SFRP2, RRAS, STC1, IGFBP5
GO:0001558	Regulation of cell growth	13	0.001477124	NOV, PRKCQ, SGK1, WISP2, WISP1, CTGF, KAZALD1, FBLN5, FOXM1, RASGRP2, IGFBP6, CEACAM1, IGFBP5
GO:0055015	Ventricular cardiac muscle cell development	5	0.001518037	CCNB1, CDK1, HEY2, LMNA, FHL2
GO:0090027	Negative regulation of monocyte chemotaxis	4	0.001571138	NOV, MINOS1-NBL1, GREM1, SLIT2
GO:0070830	Bicellular tight junction assembly	8	0.001582947	OCLN, ACTN4, MARVELD2, CLDN3, MARVELD3, CRB3, ECT2, GRHL2
GO:0050679	Positive regulation of epithelial cell proliferation	11	0.001599078	NOG, OSR1, NME1-NME2, ID1, DLX6, DLX5, FGF10, ESRP2, PAX2, SOX9, IHH
GO:0002576	Platelet degranulation	15	0.001650782	SELP, ACTN4, PDGFA, ACTN1, ECM1, TIMP3, FLNA, CTSW, ORM1, CD36, LEFTY2, SERPINA3, SERPINA1, THBS1, ORM2
GO:0016055	Wnt signaling pathway	22	0.001694244	NKD1, SPIN1, FERMT2, TLE2, FRZB, SLC9A3R1, APCDD1, WNT2B, CCNE1, RNF43, DKK3, WNT4, WISP1, RSPO1, CPE, DACT3, SFRP2, RSPO3, KREMEN1, RNF138, TGFB1I1, LRP4
GO:0045216	Cell–cell junction organization	6	0.002445448	OCLN, LIMS2, MARVELD2, MARVELD3, NLGN4X, CXADR
GO:0030308	Cell growth negative regulation	16	0.002889171	PTPRJ, CRYAB, FHL1, FBP1, OSGIN2, FRZB, GREM1, SLIT2, RERG, NOV, AGTR2, MSX1, SFRP2, DACT3, CDKN2AIP, SOX17
GO:0030514	Negative regulation of BMP signal transduction pathway	9	0.003034824	RBPMS2, CAV1, NOG, CHRDL1, DKK1, MINOS1-NBL1, SFRP2, GREM1, TOB1

**Table 3:** Kyoto Encyclopedia of Genes and Genomes pathway analysis of 1,093 differentially expressed genes between thin endometrium and adjacent normal endometrium.

**Term**		**Count**	***P*-value**	**Genes**
hsa04270	Vascular smooth muscle contraction	22	2.32E-06	KCNMA1, ACTA2, CALD1, MRVI1, PRKG1, ITPR3, KCNMB1, ITPR1, PRKCB, MYL9, ITPR2, PRKCQ, ACTG2, PLA2G4A, PLA2G2A, AVPR1A, PLA2G4F, CACNA1C, RAMP1, MYLK, ADRA1D, PPP1R14A
hsa04512	Extracellular matrix–receptor interaction	16	1.06E-04	ITGA11, COL4A6, COL4A5, HMMR, LAMA2, ITGA9, LAMB3, SDC1, CD36, ITGB8, LAMC3, COMP, ITGA7, LAMC2, THBS1, SPP1
hsa05410	Hypertrophic cardiomyopathy	15	1.14E-04	ITGA9, ACE, ACTC1, DES, TNNC1, ITGB8, DMD, ITGA7, ITGA11, LMNA, CACNB2, TPM2, CACNA1C, TPM1, SGCA
hsa04510	Focal adhesion	26	2.73E-04	CAV2, CAV1, ACTN4, PDGFA, MET, ITGA11, ACTN1, BIRC3, FLNC, COL4A6, FLNA, COL4A5, PRKCB, MYL9, LAMA2, ITGA9, LAMB3, LAMC3, ITGB8, COMP, ILK, ITGA7, LAMC2, THBS1, MYLK, SPP1
hsa04530	Tight junction	14	0.001241795	CLDN7, OCLN, CLDN4, ACTN4, CLDN3, CRB3, ACTN1, LLGL2, MYL9, CGN, MYH11, AFDN, MYH14, TJP3
hsa04514	Cell adhesion molecules	18	0.002948225	SELP, CLDN7, OCLN, CLDN4, CADM1, CLDN3, VTCN1, CD276, CDH1, HLA-DMB, ALCAM, NCAM1, ITGA9, SDC1, ITGB8, NLGN4X, VCAN, HLA-DOB
hsa04750	Inflammatory mediator regulation of transient receptor potential channels	14	0.003685506	IL1R1, CYP2J2, CAMK2G, F2RL1, ITPR3, ITPR1, PRKCB, ITPR2, PRKCQ, PLA2G4A, MAPK13, PLA2G4F, CAMK2A, MAP2K6
hsa04610	Complement and coagulation cascades	11	0.005535681	C7, CD55, MASP1, C4A, C4B, C3, F3, SERPINA5, SERPINA1, CFI, C4BPA
hsa04670	Leukocyte transendothelial migration	14	0.013940458	CLDN7, OCLN, CLDN4, ACTN4, CLDN3, ACTN1, CXCL12, PRKCB, MYL9, CTNNA2, EZR, CXCR4, MAPK13, AFDN
hsa04730	Long-term depression	9	0.020179859	GNAZ, PLA2G4A, GRIA2, PLA2G4F, PRKG1, ITPR3, ITPR1, PRKCB, ITPR2
hsa04911	Insulin secretion	11	0.022698704	KCNMA1, CAMK2G, ADCYAP1R1, SLC2A1, KCNN2, ITPR3, CACNA1C, CAMK2A, SNAP25, KCNMB1, PRKCB
hsa04020	Calcium signal transduction pathway	18	0.02706823	PTGER3, TNNC1, ERBB3, CAMK2G, ITPKB, PTGFR, ITPR3, ITPR1, PRKCB, ITPR2, GNAL, PLN, AVPR1A, CACNA1H, CACNA1C, CAMK2A, MYLK, ADRA1D
hsa00512	Mucin type O-glycan biosynthesis	6	0.029788794	GALNT3, GCNT3, GALNT4, GALNT18, GALNT12, ST6GALNAC1
hsa05150	*Staphylococcus aureus* infection	8	0.033164245	SELP, MASP1, C4A, C4B, C3, CFI, HLA-DMB, HLA-DOB
hsa04720	Long-term potentiation	9	0.033723842	GRIA2, RPS6KA1, CAMK2G, ITPR3, CACNA1C, CAMK2A, ITPR1, PRKCB, ITPR2
hsa04912	GnRH signal transduction pathway	11	0.034509876	PLA2G4A, MAPK13, CAMK2G, PLA2G4F, ITPR3, CACNA1C, CAMK2A, MAP2K6, ITPR1, PRKCB, ITPR2
hsa04115	p53 signal transduction pathway	9	0.036464983	CCNB1, CCNE1, CDK1, CCNB2, MDM4, SFN, THBS1, PERP, GTSE1
hsa04520	Adherens junction	9	0.04891771	PTPRJ, ACTN4, SORBS1, MET, ACTN1, CDH1, AFDN, NECTIN4, CTNNA2

### Genome-Wide Patterns of the miRNA Transcriptomic Landscape

First, clean reads were mapped to the human genome, and then those mapped reads were further matched to miRbase (V22). Notably, 7,004,583 reads (TE sample) and 5,717,874 reads (AJ-CN sample) were aligned to human pre-miRNAs. A total of 1,244 known miRNAs were altogether identified in our endometrial samples. According to the results of the miRNA-seq analysis, 72 known miRNAs were deemed to be DEMs between the thin adhesive endometrium of the IUA group and the control adjacent normal endometrial cells. Among these DEMs, five miRNAs were upregulated and 67 were downregulated compared with the control adjacent normal endometrial cells (Supplementary Table 4). The five upregulated and top 10 downregulated DEMs are shown in Table 4.

**Table 4:** The five upregulated and top 10 downregulated differentially expressed miRNAs in thin endometrium.

**MATURE-ID**	**PRE-ID**	**MATURE-SEQ**	**Log2 (fold change)**
hsa-miR-1-3p	hsa-mir-1-2	UGGAAUGUAAAGAAGUAUGUAU	4.369546
hsa-miR-133a-3p	hsa-mir-133a-1	UUUGGUCCCCUUCAACCAGCUG	3.602664
hsa-miR-143-3p	hsa-mir-143	UGAGAUGAAGCACUGUAGCUC	2.285041
hsa-miR-133b	hsa-mir-133b	UUUGGUCCCCUUCAACCAGCUA	2.142958
hsa-miR-145-5p	hsa-mir-145	GUCCAGUUUUCCCAGGAAUCCCU	1.896256
hsa-miR-34c-5p	hsa-mir-34c	AGGCAGUGUAGUUAGCUGAUUGC	−6.13482
hsa-miR-200a-3p	hsa-mir-200a	UAACACUGUCUGGUAACGAUGU	−5.88598
hsa-miR-200c-3p	hsa-mir-200c	UAAUACUGCCGGGUAAUGAUGGA	−5.57759
hsa-miR-200b-3p	hsa-mir-200b	UAAUACUGCCUGGUAAUGAUGA	−5.57684
hsa-miR-375	hsa-mir-375	UUUGUUCGUUCGGCUCGCGUGA	−5.49063
hsa-miR-449c-5p	hsa-mir-449c	UAGGCAGUGUAUUGCUAGCGGCUGU	−5.24102
hsa-miR-429	hsa-mir-429	UAAUACUGUCUGGUAAAACCGU	−5.17173
hsa-miR-141-3p	hsa-mir-141	UAACACUGUCUGGUAAAGAUGG	−5
hsa-miR-449a	hsa-mir-449a	UGGCAGUGUAUUGUUAGCUGGU	−4.92875
hsa-miR-182-5p	hsa-mir-182	UUUGGCAAUGGUAGAACUCACACU	−4.89552
hsa-miR-34c-5p	hsa-mir-34c	AGGCAGUGUAGUUAGCUGAUUGC	−6.13482
hsa-miR-200a-3p	hsa-mir-200a	UAACACUGUCUGGUAACGAUGU	−5.88598
hsa-miR-200c-3p	hsa-mir-200c	UAAUACUGCCGGGUAAUGAUGGA	−5.57759
hsa-miR-200b-3p	hsa-mir-200b	UAAUACUGCCUGGUAAUGAUGA	−5.57684
hsa-miR-375	hsa-mir-375	UUUGUUCGUUCGGCUCGCGUGA	−5.49063
hsa-miR-449c-5p	hsa-mir-449c	UAGGCAGUGUAUUGCUAGCGGCUGU	−5.24102
hsa-miR-429	hsa-mir-429	UAAUACUGUCUGGUAAAACCGU	−5.17173
hsa-miR-141-3p	hsa-mir-141	UAACACUGUCUGGUAAAGAUGG	−5
hsa-miR-449a	hsa-mir-449a	UGGCAGUGUAUUGUUAGCUGGU	−4.92875

**Figure 3:**
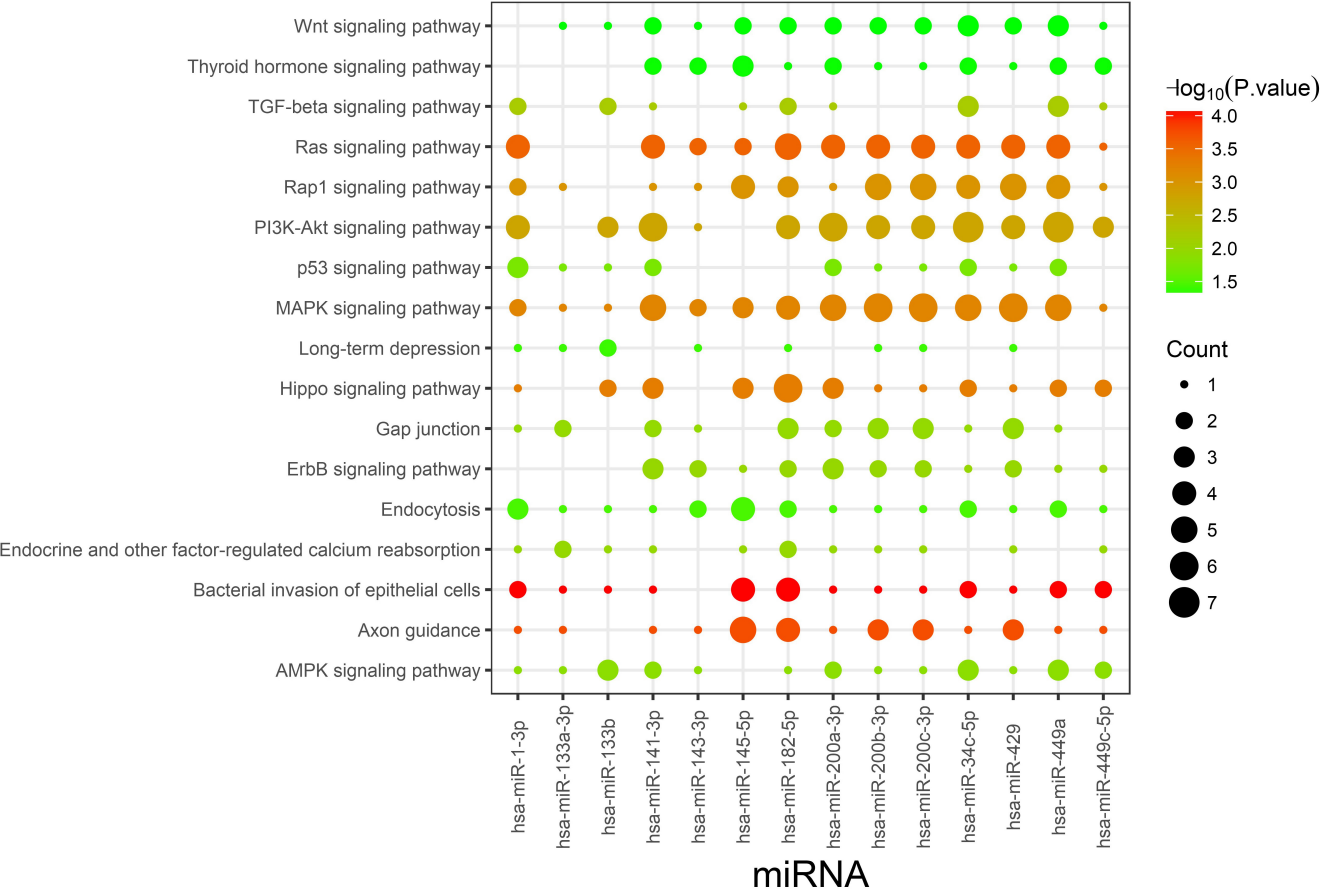
Kyoto Encyclopedia of Genes and Genomes pathway enrichment analysis of the identified targets of differentially expressed miRNAs. Count indicates the number of predicted target genes.

TargetScan and miRDB were used to characterize the putative target mRNAs of the 72 candidate DEMs in thin endometrium and to better illustrate the functions of DEMs. TargetScan and miRDB were employed to identify 812 common candidate target genes for the 15 DEMs (Supplementary Table 5). Then, GO and KEGG analyses were performed for the 812 target genes. GO enrichment analyses suggested that the target genes of multiple DEMs were associated with the regulation of angiogenesis, MAPK activation, negative regulation of cell migration, negative regulation of stress fiber assembly, positive regulation of epithelial cell proliferation, regulation of the canonical Wnt signaling pathway, and positive regulation of cell proliferation (Table 5 and Supplementary Figure 1). The KEGG pathways in which the DEM targeted genes are involved were discovered, which included the Ras signal transduction pathway, Hippo signal transduction pathway, MAPK signal transduction pathway, PI3K–Akt signal transduction pathway, gap junction, p53 signaling pathway, Wnt signal transduction pathway, and ErbB signal transduction pathway (Figure 3 and Table 6). The PI3K/Akt signal transduction pathway is suggested to participate in endometrial regeneration induced by granulocyte macrophage–CSF therapy (Liu et al., 2020).

**Table 5:** Gene Oncology analysis of the identified targets of differentially expressed miRNAs between thin endometrium and adjacent normal endometrium.

**Term**		**Count**	**Fold enrichment**	***P*-value**	**Targeted genes**
GO:0007264	Small GTPase-mediated signal transduction	22	2.37	3.79E-04	RALGPS2, RAB3C, RAP2C, RAP1GDS1, RASGEF1B, RHOQ, ARF6, PLCE1, RAB43, ARF3, ARF4, ARHGAP1, YWHAQ, RAB5A, RAB14, RRAS, RHEB, RAB6B, RAP1B, RAB38, RIT2, RAB21
GO:0045944	Positive regulation of transcription from RNA polymerase II promoter	43	1.70	8.26E-04	FOSL2, HELZ2, LMO4, EDN1, RHOQ, INO80, EGLN1, PAX3, ZEB1, ASH2L, PAX7, RARB, PPP3CA, MYC, GABPB2, SATB2, RARG, KLF12, EPAS1, MET, EOMES, IGF1, DLL1, DDX5, NCL, TET1, RBMX, BCL2L12, RNF222, FOXP1, PPARGC1B, MYCN, ASCL1, RPS6KA4, EBF3, ETS1, SP3, JUN, ARF4, ZFPM2, TFAP2D, NR5A2, BMPR1B
GO:0045765	Regulation of angiogenesis	6	7.24	1.02E-03	ETS1, EFNA1, EGLN1, EMP2, VASH2, VASH1
GO:0000045	Autophagosome assembly	8	4.69	1.28E-03	GABARAPL2, GABARAPL1, ATG4B, MAP1LC3B, TRAPPC8, RB1CC1, WIPI2, TP53INP2
GO:0000187	Activation of MAPK activity	8	4.00	3.32E-03	MAP3K7, PLCE1, NTF3, EFNA1, IGF1, LPAR1, THBS1, FRS2
GO:0030336	Negative regulation of cell migration	10	3.20	3.64E-03	DLC1, RECK, TMEFF2, PTPRK, RAP2C, CLIC4, SULF1, RRAS, SRGAP1, SRGAP2
GO:0051592	Response to calcium ion	6	5.13	5.29E-03	CAV1, SLC25A13, ALG2, AHCYL1, PPP3CA, THBS1
GO:0000422	Mitophagy	6	4.39	1.04E-02	GABARAPL2, GABARAPL1, ATG4B, MAP1LC3B, RB1CC1, WIPI2
GO:0051497	Negative regulation of stress fiber assembly	4	6.31	2.28E-02	DLC1, TMEFF2, ARHGAP6, PPFIA1
GO:0016601	Rac protein signal transduction	4	6.31	2.28E-02	EPS8, WASF1, ELMO1, NCKAP1
GO:0050679	Positive regulation of epithelial cell proliferation	6	3.51	2.61E-02	WDR48, NOTCH1, FGF9, IGF1, MYC, FOXP1
GO:0008277	Modulation of G-protein coupled receptor protein signal transduction pathway	4	5.86	2.80E-02	PLCE1, GPR158, KCTD16, USP33
GO:0060828	Modulation of canonical Wnt signal transduction pathway	4	5.47	3.37E-02	AMER1, CCNY, CTNND2, CDK14
GO:0035556	Intracellular signal transduction	22	1.60	3.52E-02	ARHGEF3, SGK1, NUAK1, PREX1, DSTYK, SPSB4, ITSN1, PLCL2, RPS6KA4, SNRK, DGKE, PPP1R1C, STAC, GUCY1A3, DGKZ, RGS7, STK39, DCX, STK38L, PAG1, NET1, SHC4
GO:0007205	Protein kinase C-activating G-protein coupled receptor signaling pathway	4	5.13	4.01E-02	GRM5, DGKE, EDN1, DGKZ
GO:0008284	Positive regulation of cell proliferation	18	1.68	4.05E-02	RARG, PDCD10, NTF3, PTH1R, IGF1, DLL1, PTGFR, TET1, TGFB2, CRKL, KRAS, ASH2L, HBEGF, RARB, MAB21L1, EMP2, CSF1R, SHC4

**Table 6:** Kyoto Encyclopedia of Genes and Genomes pathway analysis of the identified targets of differentially expressed miRNAs between thin endometrium and adjacent normal endometrium.

**Term**		**Count**	**Fold enrichment**	***P*-value**	**Targeted genes**
cfa05100	Bacterial invasion of epithelial cells	13	3.92	9.32E-05	ACTB, CAV1, CLTA, WASF1, MET, CLTC, CD2AP, ELMO1, ACTG1, CTTN, CRKL, GAB1, SHC4
cfa04360	Axon guidance	16	3.08	1.86E-04	GNAI3, EFNA1, MET, NTNG1, L1CAM, EPHA2, SLIT2, SEMA6A, KRAS, CFL2, CFL1, SEMA3A, PPP3CA, RASA1, SRGAP1, SRGAP2
cfa04014	Ras signaling pathway	22	2.41	2.81E-04	FGF9, GRB2, EFNA1, MET, IGF1, ARF6, EPHA2, KDR, PLCE1, KRAS, ETS1, GAB1, PDGFRA, RAB5A, RAPGEF5, RRAS, RAP1B, PRKACB, ABL2, RASA1, CSF1R, SHC4
cfa04390	Hippo signaling pathway	17	2.69	5.28E-04	ACTB, MOB1B, YWHAZ, MPP5, LEF1, SMAD1, TGFB2, AJUBA, ACTG1, YWHAG, CCND2, PPP2CA, PPP2CB, YWHAQ, BMPR1B, MYC, FBXW11
cfa04010	MAPK signaling pathway	23	2.21	6.38E-04	LAMTOR3, NTF3, FGF9, GRB2, MAP2K4, CACNB3, CACNB4, TGFB2, MAP3K7, BDNF, CRKL, KRAS, RPS6KA4, DUSP1, JUN, PDGFRA, RRAS, RAP1B, PRKACB, PPP3CA, MYC, RASA1, DUSP6
cfa04015	Rap1 signaling pathway	20	2.30	9.89E-04	ACTB, GNAI3, FGF9, EFNA1, MET, IGF1, LPAR1, EPHA2, KDR, ACTG1, PLCE1, CRKL, KRAS, GNAQ, PDGFRA, RAPGEF5, RRAS, RAP1B, THBS1, CSF1R
cfa04151	PI3K–Akt signaling pathway	27	1.91	1.71E-03	YWHAZ, PPP2R3A, EFNA1, GRB2, FGF9, LPAR1, FOXO3, CCNE2, KRAS, PPP2CA, PPP2CB, PIK3AP1, THBS1, MYC, CSF1R, SGK1, MET, IGF1, IL6R, EPHA2, KDR, YWHAG, EIF4E, CCND2, YWHAQ, PDGFRA, RHEB
cfa04350	TGF-beta signaling pathway	10	2.94	6.36E-03	E2F5, PPP2CA, PPP2CB, TGIF2, SMAD1, SKP1, THBS1, BMPR1B, MYC, TGFB2
cfa04012	ErbB signaling pathway	10	2.74	1.01E-02	CRKL, KRAS, GRB2, JUN, GAB1, MAP2K4, HBEGF, MYC, ABL2, SHC4
cfa04961	Endocrine and other factor-regulated calcium reabsorption	7	3.71	1.04E-02	CLTA, AP2B1, ATP1B3, GNAQ, PTH1R, PRKACB, CLTC
cfa04540	Gap junction	10	2.71	1.08E-02	GRM5, GNAI3, KRAS, GNAQ, GRB2, PDGFRA, GJA1, GUCY1A3, PRKACB, LPAR1
cfa04152	AMPK signaling pathway	12	2.35	1.27E-02	MAP3K7, PPP2R3A, HNF4A, PFKFB3, PPP2CA, PPP2CB, RAB14, ADIPOR2, IGF1, RHEB, FOXO3, SCD5
cfa04115	p53 signaling pathway	8	2.94	1.81E-02	CCNE2, CCND2, ZMAT3, SHISA5, IGF1, MDM4, THBS1, SESN1
cfa04144	Endocytosis	17	1.74	3.41E-02	CAV1, CLTA, PSD3, VPS37B, ARF6, SNX4, ASAP3, CLTC, DAB2, AP2B1, CHMP1A, ARF3, PDGFRA, RAB5A, GIT2, STAM, EPN1
cfa04730	Long-term depression	7	2.78	3.82E-02	GNAI3, KRAS, GNAQ, PPP2CA, PPP2CB, GUCY1A3, IGF1
cfa04919	Thyroid hormone signal transduction pathway	10	2.11	4.61E-02	ACTB, ACTG1, SLC16A2, PLCE1, NOTCH1, KRAS, ATP1B3, RHEB, PRKACB, MYC
cfa04310	Wnt signal transduction pathway	11	2.00	4.69E-02	MAP3K7, CTBP2, CCND2, JUN, LEF1, PRKACB, PPP3CA, SKP1, DAAM1, MYC, FBXW11

### DEG–DEM Regulatory Network and Functional Assessment

**Figure 4:**
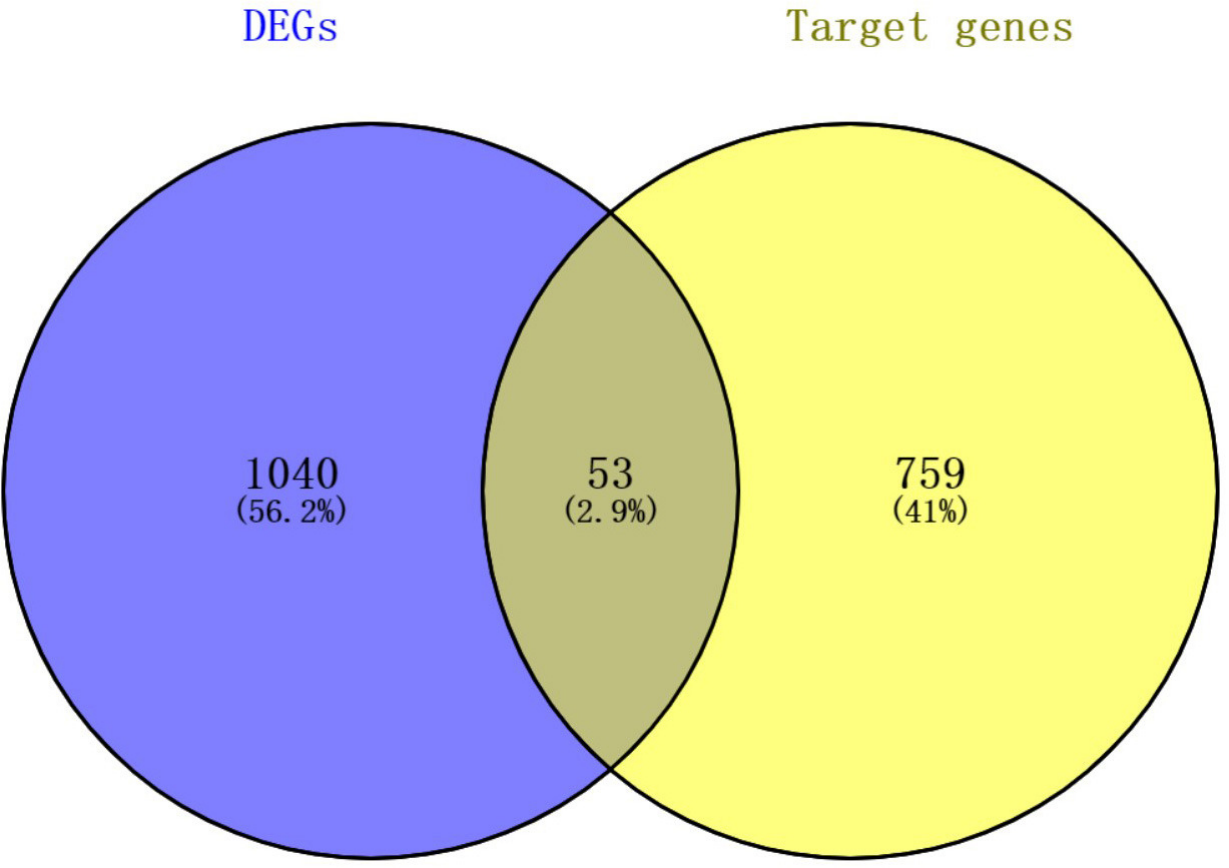
The intersecting mRNAs between the common predicted target mRNAs and differentially expressed genes.

**Figure 5:**
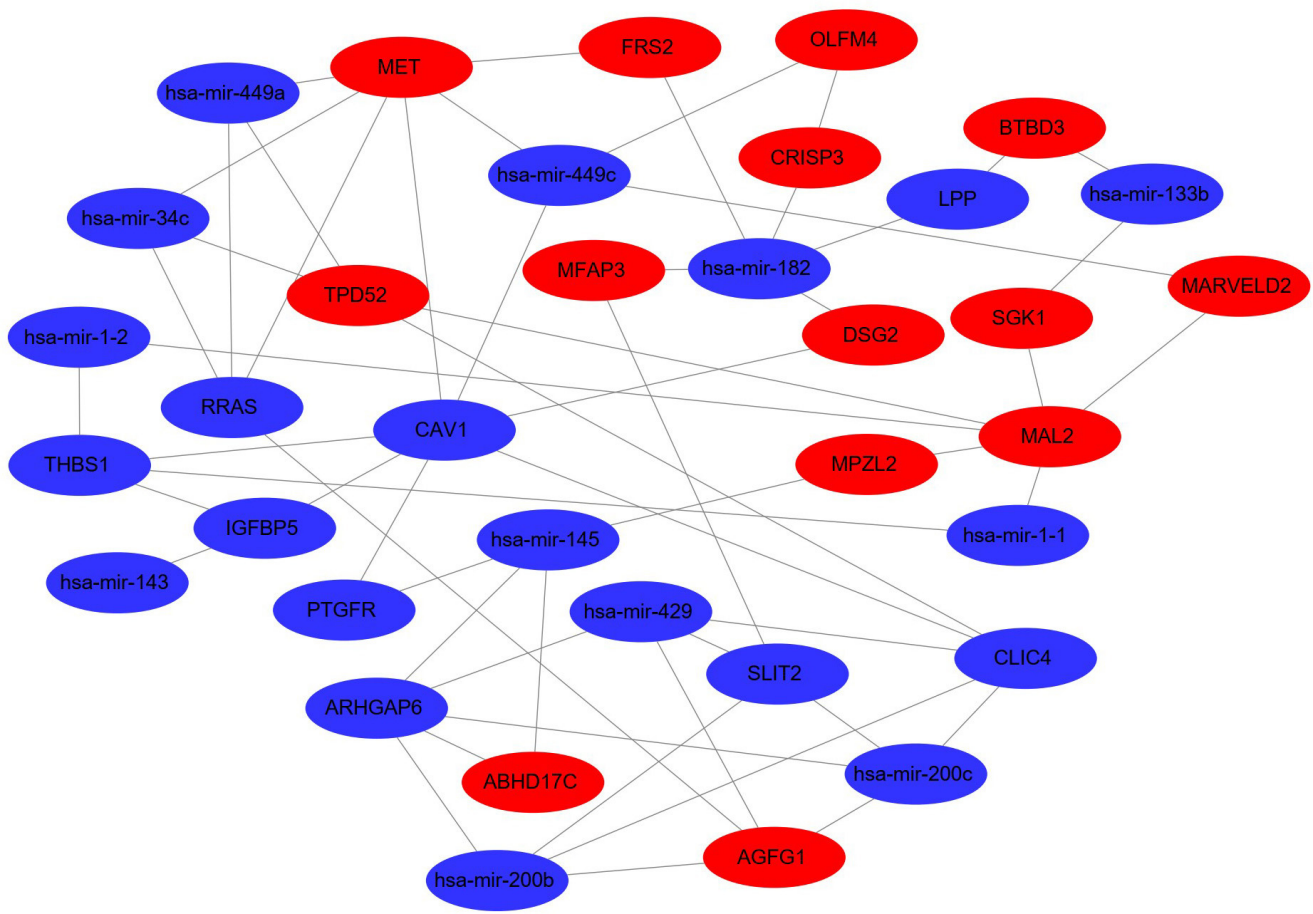
Differentially expressed miRNA–differentially expressed gene regulatory network. The red and green colors denote upregulation and downregulation, respectively.

For the establishment of the DEG-DEM regulatory network, 53 (21 upregulated genes and 32 downregulated genes) overlapping genes were discovered by comparing the target genes of DEMs (five were upregulated and 10 were downregulated) with DEGs, and they were deemed as consistently expressed genes (CEGs) (Figure 4). The STRING database was used to construct the PPI network using the CEG list. As shown in Figure 5, CAV1, MET, MAL2, has-mir-138, ARHGAP6, CLIC4, RRAS, AGFG1, has-mir-200, and has-mir-429 were the top 10 hub genes that interacted with the maximum number of nodes. Additionally, the gene pathway–function interactions were analyzed, and the identified hub genes showed significant enrichment in negative regulation of cell growth and inflammatory response regulation. For a better assessment of how this miRNA–mRNA regulatory network affected thin endometrium, a KEGG pathway analysis of CEGs was performed. The miRNA-mediated gene regulatory network in thin endometrium plays important roles in the regulation of the FoxO signaling pathway and the regulation of autophagy (Table 7).

**Table 7:** Gene Ontology and Kyoto Encyclopedia of Genes and Genomes pathway enrichment analyses of consistently expressed genes.

**Term**	**Count**	**Fold enrichment**	***P*-value**	**Genes**
GO:0030308—cell growth negative regulation	2	76.69298	0.025132	OSGIN2, SLIT2
GO:0006954—inflammatory response	3	10.30204	0.032433	SGK1, THBS1, PTGFR
GO:0001558—cell growth regulation	2	20.00686	0.093047	SGK1, IGFBP5
GO:0007605—sensory perception of sound	2	20.00686	0.093047	CLIC4, MARVELD2
xtr04068: FoxO signaling pathway	3	6.969466	0.059369	SGK1, GABARAPL1
xtr04140: regulation of autophagy	2	24.34667	0.074102	GABARAPL1

## Discussion

IUA, which is characterized by endometrial fibrosis and thin endometrium, was always regarded as a major cause of female infertility and a major challenge to clinical therapy. Even through a surgical operation combined with hormone treatment, TE with severe endometrial injuries is difficult to restore. The previous transcriptomic microarray analysis discovered 318 upregulated genes and 322 downregulated genes in thin endometrium and revealed the abnormal activation of the inflammatory environment and an abnormal decrease in the OS response in thin endometrium (Maekawa et al., 2017). Current knowledge about the pathogenesis and involvement of miRNA–mRNA networks in thin endometrium is limited. In this study, gene expression patterns of thin endometrium along with the matched control endometrial tissues from women were explored, and we revealed the abnormal activation of the inflammatory environment and an abnormal decrease in the OS response in thin endometrium. To our knowledge, this study is the first to employ self-controlled transcriptomic analysis to investigate the regulatory functions of miRNA–mRNA networks of cells from the mid-secretory thin endometrium and adjacent normal endometrial cells.

As revealed in our results, some genes were abnormally expressed at the time of disease onset, revealing that thin endometrium may have occurred as a type of endometrial disorder due to the abnormal expression of genes within endometrial tissues prior to lesion occurrence. Indeed 1,093 genes were significantly differentially expressed in thin endometrium. A total of 74 DEGs associated with TE in our study were consistent with a previous study performed in thin and control endometrial samples using a microarray (Maekawa et al., 2017), including those that were up-regulated. Furthermore, our DEG functional enrichment analysis also revealed the involvement of angiogenesis and negative regulation of growth and cell migration in thin endometrium. Typically, during each menstrual cycle, angiogenesis promotes new blood vessel formation and is crucial for endometrial regeneration by supplying a vascularized and receptive endometrium for embryo implantation. Previous studies also show that the vascular endothelial growth factor (VEGF) could be a regulator of endometrial angiogenesis. Thus, the differential expression of VEGF and the blockade of angiogenesis in our study could be considered as pathological changes of the scarred thin endometrium (Jiang et al., 2019).

Interestingly, consistent with previous miRNA expression profiles reported for the recurrent implantation failure endometrium (Vilella et al., 2015; Rekker et al., 2018), some upregulated DEMs in TE in our study also belonged to the miR-200 family, including miR-200a-3p, miR-200c-3p, miR-200c-5p, miR-141-3p, and miR-429. The miR-200 family has been suggested to target multiple genes that are involved in cell proliferation, invasion, and inflammation. Thus, the aberrant expression of miR-200 may negatively regulate the endometrial development which would result in endometriosis or endometrial cancer (Panda et al., 2012).

Through analyzing the interactions between DEMs and their targets, some vital pathways, including MAPK, p53, PI3K–Akt, and Wnt signal transduction, were found to participate in TE. As endometrial thickness has been recognized as an important indicator of endometrial receptivity (Ledee-Bataille et al., 2002), we thus assume that the abnormalities of these pathways may compromise the development of the endometrium. For example, rapid activation of PI3K/Akt signaling cascades by growth factors and estrogen is involved in the migration of normal endometrial stromal cells (Gentilini et al., 2007). However, the expression of DEGs in the PI3K/AKT pathway, including EFNA1, FGF9, LPAR1, CCNE2, SGK1, MET, IL6R, and PDGFRA, was decreased in thin endometrium, which suggests that the repair ability of the thin endometrium was impaired during the proliferative phase (Le et al., 2016). Similarly, the abnormal Wnt/beta-catenin signal pathway would also impair the proliferation of estrogen-dependent endometrial cells (Tepekoy et al., 2015).

In the present study, our miRNA–mRNA regulatory networks provided a complete profile for the underlying mechanism of thin endometrium formation, and the hub genes identified in the networks may play certain roles in the development of thin endometrium. CAV1 expression is associated with cell survival and proliferation (Zhao et al., 2013). MET, the receptor for insulin-like growth factor, potentially affects the functions of the endometrium (Satterfield et al., 2008). Therefore, the present study may provide useful information for understanding of the miRNA-mediated changes in mRNA expression in thin endometrium, and a further understanding of the functions of **miRNA–mRNA** networks can provide a new perspective for future studies examining potentially novel biomarkers and therapeutic targets.

## Data Availability Statement

The datasets presented in this study can be found in online repositories. The names of the repository/repositories and accession number(s) can be found in the article/Supplementary Materials.

## Ethics Statement

The studies involving human participants were reviewed and approved by the Ethics Committees on Human Research of the First Hospital Affiliated with University of Science and Technology of China. The patients/participants provided their written informed consent to participate in this study.

## Author Contributions

DL, ZW, and WT contributed to sample collection. XT and YM designed the experiment. SZ, LZ, and BX performed the experiment, data analysis, and manuscript preparation. XT and BX revised the manuscript. All authors contributed to the article and approved the submitted version.

## Conflict of Interest

The authors declare that the research was conducted in the absence of any commercial or financial relationships that could be construed as a potential conflict of interest.
