# Supplementary material for: Integrated Transcriptomic Analysis of the miRNA–mRNA Interaction Network in Thin Endometrium
Source: Front Genet. 2021 Mar 16;12:589408. doi: 10.3389/fgene.2021.589408 (PMC8009322; doi:10.3389/fgene.2021.589408)

**Integrated transcriptome analysis of miRNA-mRNA interaction network in thin endometrium**

Shengxia Zheng^1#^, Lu Zong^1#^, Ye Meng^1^, Daojing Li^1^, Zhenyun Wang^1^, Xianhong Tong^1^, Bo Xu^1*^

1. Center for Reproductive Medicine, The First Affiliated Hospital of USTC, Division
of Life Sciences and Medicine, University of Science and Technology of China, Hefei 230001, P.R. China

^#^These authors contributed equally to this study

*****To whom correspondence should be addressed. E-mail: Bo Xu: bioxubo@mail.ustc.edu.cn

#### Supplementary Figure1. GO analysis of predicted targets of differentially expressed miRNAs
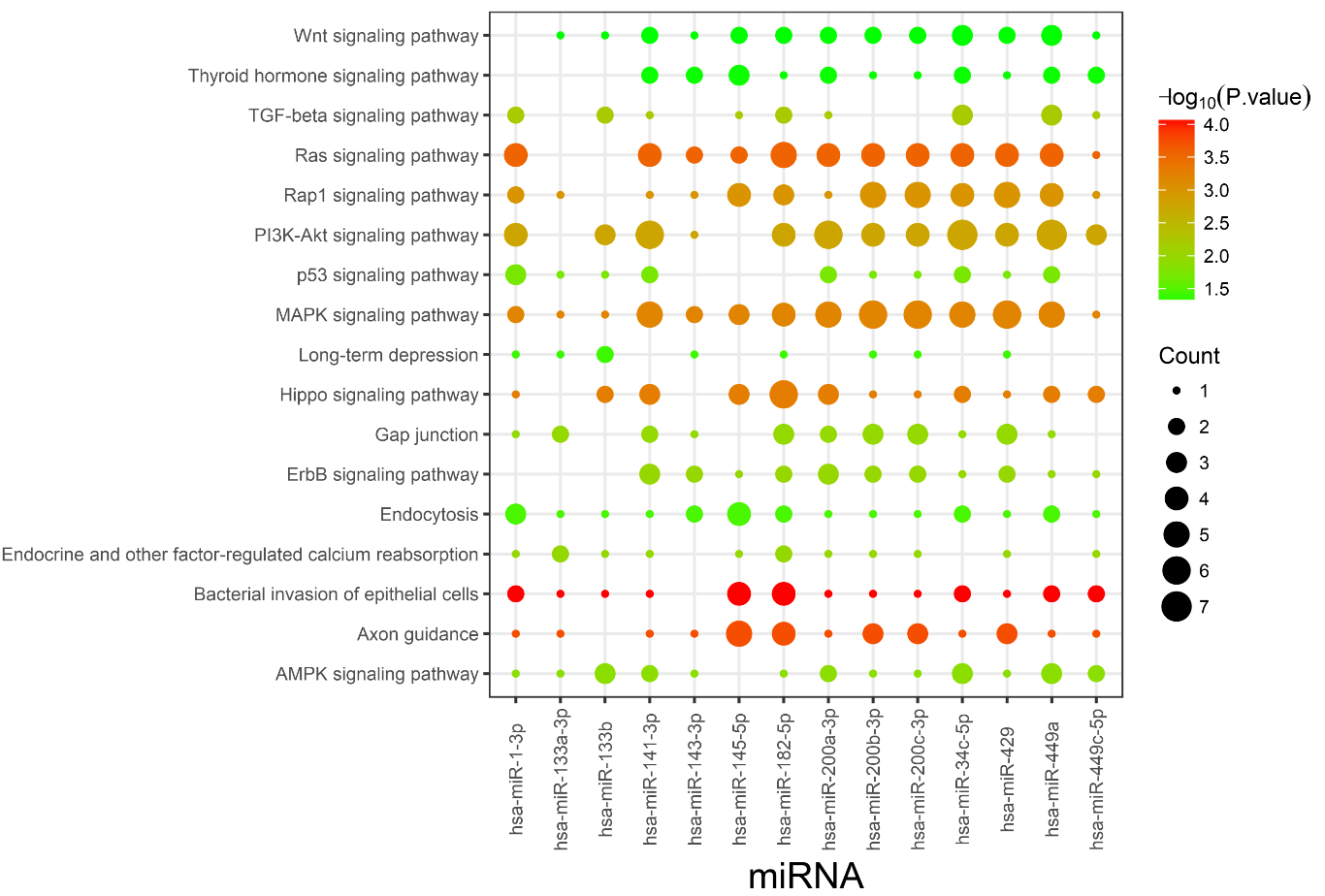

Supplement: Supplementary file 6 [file Data_Sheet_1.docx]
